# Exploring celecoxib polymorph landscape using AIMNet2 machine learning interatomic potential

**DOI:** 10.1039/d5sc09784c

**Published:** 2026-06-10

**Authors:** Peikun Zheng, Yuriy A. Abramov, Changquan Calvin Sun, Olexandr Isayev

**Affiliations:** a Department of Chemistry, Carnegie Mellon University Pittsburgh Pennsylvania 15213 USA olexandr@olexandrisayev.com; b Porton USA| J-Star Research Inc Cranbury NJ 08512 USA; c Eshelman School of Pharmacy, University of North Carolina Chapel Hill NC 27599 USA; d Department of Industrial and Molecular Pharmaceutics, Purdue University 124C RHPH, 575 Stadium Mall Drive West Lafayette IN 47907 USA

## Abstract

The crystal form a drug adopts can change everything from how it dissolves to whether it works in the clinic, yet predicting which polymorphs a flexible molecule will produce remains one of the most stubborn problems in pharmaceutical science. Competing forms typically differ in energy by less than 2 kJ mol^−1^, a precision that quantum chemistry can reach only at forbidding cost. Here we deploy AIMNet2, a machine-learned interatomic potential refined by active learning on cluster reference data, to map the polymorphic landscape of celecoxib, a widely prescribed COX-2 inhibitor whose form I exhibits record-breaking elastic flexibility. A GPU-accelerated workflow generates and ranks hundreds of thousands of candidate structures at near-quantum accuracy, recovers the experimental ordering of forms I, II, and III with sub-Ångström geometric fidelity, and identifies two low-energy candidate structures within 4 kJ mol^−1^ of the most stable known polymorph. Hybrid-DFT calculations yield a similar low-energy landscape in which multiple polymorphs remain thermodynamically competitive. Finite-temperature analyses further expose the limits of static-lattice models for ultra-soft crystals such as form I. Beyond celecoxib, the framework supplies physically motivated targets for experimental polymorph screening and a transferable strategy for crystal-structure prediction across flexible drug molecules.

## Abstract 2

Active-learning-refined AIMNet2 accurately predicts celecoxib polymorphs, reproducing forms I–III, identifying new low-energy candidates.

## Graphical abstract

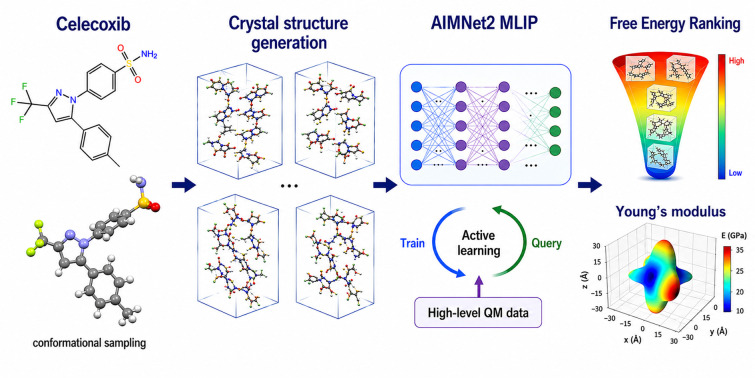

## Introduction

Polymorphism, the ability of a compound to crystallize in more than one distinct crystal structure, is a widely observed phenomenon in organic solids. It is estimated that a substantial fraction of organic molecules exhibit polymorphism in the solid state, and such variations in crystal packing can significantly impact key physicochemical and biopharmaceutical properties, including solubility, dissolution kinetics, and bioavailability. For active pharmaceutical ingredients (APIs), the identification, characterization, and control of polymorphs are critical aspects of drug development and formulation, as different polymorphic forms can exhibit distinct therapeutic performance and patentability.^1,2^

Celecoxib (CEL), a nonsteroidal anti-inflammatory drug (NSAID) and the first selective cyclooxygenase-2 (COX-2) inhibitor approved for the treatment of rheumatoid arthritis and osteoarthritis, has been extensively studied for its polymorphism due to its clinical relevance and poor aqueous solubility. To date, four polymorphic forms of celecoxib (forms I–IV) have been reported, with form III identified as the thermodynamically stable form under ambient conditions.^3^ While the crystal structure of form III was determined *via* single-crystal X-ray diffraction and deposited in the Cambridge Structural Database (CSD) in 1999,^4^ the structure of form II was not reported until 2019.^5^ Due to challenges in obtaining suitable single crystals, form I crystal structure was not solved until 2025 when sufficiently large single crystals were grown through melt crystallization, revealing an unprecedented elastic strain exceeding 8.7%, setting a new record for organic molecular crystals.^6,7^ Form IV remains structurally uncharacterized. These observations point to a shallow free-energy landscape with several near-isoenergetic minima, raising the prospect of additional, as-yet-uncharacterized forms. Such forms could offer improved solubility or mechanical performance, motivating systematic polymorph screening and computational prediction.

Given the growing importance of polymorph control in pharmaceutical development and the inherent difficulty of experimentally isolating all relevant forms, computational crystal structure prediction (CSP) has become an indispensable tool for mapping the polymorphic landscape of drug molecules. Traditional CSP approaches, typically based on empirical force fields or density functional theory (DFT), have achieved moderate success in rationalizing known polymorphs of various pharmaceutical compounds. However, these methods face two critical challenges, particularly in complex systems such as celecoxib, which exhibit a high degree of freedom due to a combination of molecular flexibility and a large number of symmetry-independent molecules in the unit cell (*Z*′ = 3 for CEL form I):^8^ (i) the free energy differences between competing polymorphs are often extremely small (on the order of 1–2 kJ mol^−1^), making reliable energetic ranking highly sensitive and uncertain; and (ii) the high computational cost of accurate electronic structure methods, such as dispersion-corrected hybrid DFT, renders exhaustive polymorph screening computationally prohibitive. This computational bottleneck is particularly acute for large-scale screening and for systems requiring high accuracy, as demonstrated in recent CSP blind tests.^9–12^ While hierarchical schemes using force fields followed by DFT are common, the accuracy limitations of force fields and the cost of DFT remain significant hurdles.

Machine-learned interatomic potentials (MLIPs) now reproduce DFT energies and forces at orders-of-magnitude lower cost, enabling exhaustive crystal-structure sampling that is intractable with electronic-structure methods directly. Trained on high-level quantum mechanical (QM) data, ML can accurately capture subtle conformational energy differences and intermolecular forces, which are essential for distinguishing between closely related polymorphs. Furthermore, the scalability and flexibility of ML-based approaches enable efficient large-scale sampling of crystal structures, thereby facilitating comprehensive exploration of the polymorphic landscape. For instance, a hierarchical CSP workflow that combines systematic crystal packing searches with ML force fields has been validated on 66 molecules comprising 137 experimentally known polymorphs, successfully reproducing all known forms and predicting new low-energy candidates that may impact downstream development.^13^ Recent studies have demonstrated the potential of machine-learned interatomic potentials (MLIPs) in improving energy ranking efficiency in CSP workflows^14–17^ and highlighted the development of Δ-ML strategies to enhance baseline methods for describing complex interactions crucial in crystals.^18–20^ End-to-end CSP pipelines incorporating MLIPs for both structure generation and optimization have also demonstrated considerable promise in balancing accuracy and computational efficiency.^21^ Despite their rapid progress, applications of ML in pharmaceutical CSP remain limited, and their potential to resolve long-standing challenges in polymorph prediction and energy ranking remains to be fully demonstrated. The recent seventh CSP blind test further emphasized the need for more efficient and accurate methods, especially for flexible molecules and complex systems, while also highlighting the promise of system-specific MLIPs such as AIMNet2 (ref. 22) in achieving competitive accuracy with significantly reduced computational overhead.^10,11,23^

In this work, we employ a fine-tuned AIMNet2 model to systematically investigate the polymorphic landscape of celecoxib. Our approach integrates GPU-accelerated crystal structure generation, geometry optimization, and energy ranking across thousands of candidate structures. The workflow not only reproduces the experimentally observed energy ordering of known polymorphs but also predicts two energetically competitive structures with distinct packing motifs that have not been experimentally observed. In addition, it resolves temperature-dependent stability rankings and quantifies mechanical properties. This study highlights the effectiveness of AIMNet2-based CSP in complex pharmaceutical systems and offers new insights into the solid-state behavior of celecoxib.

## Results and discussion

### AIMNet2-based workflow for molecular crystal prediction

**Fig. 1:**
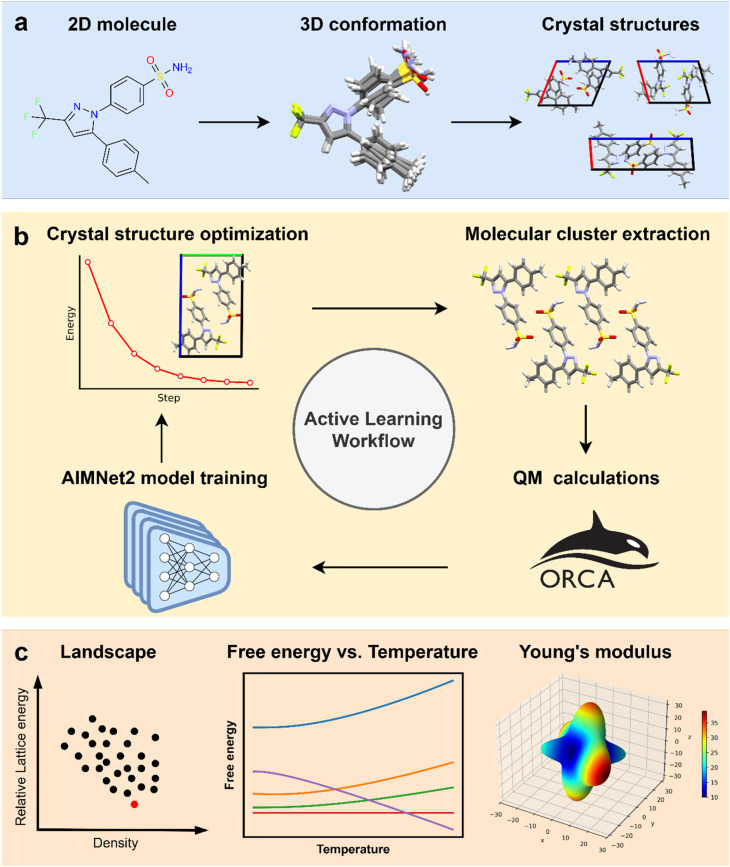
Overview of the crystal structure prediction workflow for celecoxib. (a) Crystal structure generation. (b) Active learning strategy for training the AIMNet2 model. (c) Prediction of relative lattice energy landscape at 0 K, temperature-dependent free energy, and Young's modulus using the trained AIMNet2 model.

Fig. 1 illustrates the core framework of the AIMNet2-based crystal structure prediction workflow, which consists of three main components. Additional details for each step of the workflow are provided in the SI. As shown in Fig. 1a, the process begins with the two-dimensional (2D) structure of the molecule. Reasonable three-dimensional (3D) conformers are generated using conformation generation tools, followed by the construction of candidate crystal structures based on space group symmetry, the number of molecules per unit cell (*Z*), and the number of symmetry-independent molecules (*Z*′). To enable large-scale generation of candidate structures, we have implemented a PyTorch-based crystal generator with GPU acceleration (see SI). This “2D → 3D → crystal structure” pipeline efficiently explores the polymorphic landscape of the molecule, laying the foundation for subsequent energy ranking and property prediction.

Fig. 1b illustrates the active learning strategy employed to build a high-accuracy AIMNet2 potential. We first optimize the initial crystal structures and extract representative molecular clusters from the optimized geometries. These clusters are then subjected to QM calculations at PBE + D4/def2-TZVP level of theory to obtain reference training data, which are subsequently used to fine-tune the pre-trained AIMNet2 model. This iterative data-model optimization loop not only reduces computational costs but also significantly enhances the model's generalization capability.

Finally, as illustrated in Fig. 1c, the trained AIMNet2 model enables rapid evaluation of the relative energies of a large number of candidate crystal structures, yielding a comprehensive energy landscape at 0 K. Furthermore, we perform phonon calculations to analyze the temperature-dependent Helmholtz free energy of the crystals within harmonic approximation (HA) and evaluate mechanical properties such as the Young's modulus across different polymorphs. These properties are essential for understanding the stability, processability, and pharmaceutical performance of molecular crystals.

The computational cost associated with each stage of the crystal structure prediction workflow is summarized in Table S6. Although the active learning stage involves QM evaluations for more than 20 000 *N*-mers, these calculations are performed on molecular clusters and are therefore significantly less expensive than periodic DFT calculations. Compared to traditional CSP workflows that often prune structures using low-level force fields and perform periodic DFT on only a small number of candidates, our approach reallocates computational cost toward a near-QM-quality neural network potential. As shown in Fig. S7, AIMNet2 reproduces PBE + MBD relative lattice energies with a MAE of 1.84 kJ mol^−1^ across the candidate crystal structures. Given that energy differences between competing pharmaceutical polymorphs are often on the order of 1–2 kJ mol^−1^, this level of agreement indicates that AIMNet2 captures the overall energetic landscape sufficiently well for large-scale screening and preliminary ranking of crystal structures, while retaining a computational cost that is orders of magnitude lower than periodic DFT calculations.

This strategy enables the refinement and ranking of tens of thousands of fully flexible crystal structures while minimizing the risk of prematurely discarding physically relevant candidates. Overall, although the workflow does not eliminate the need for DFT calculations, it substantially reduces the number of expensive periodic calculations and provides a more faithful exploration of the crystal energy landscape at a computational cost that remains tractable.

### Polymorphic energy landscapes

**Fig. 2:**
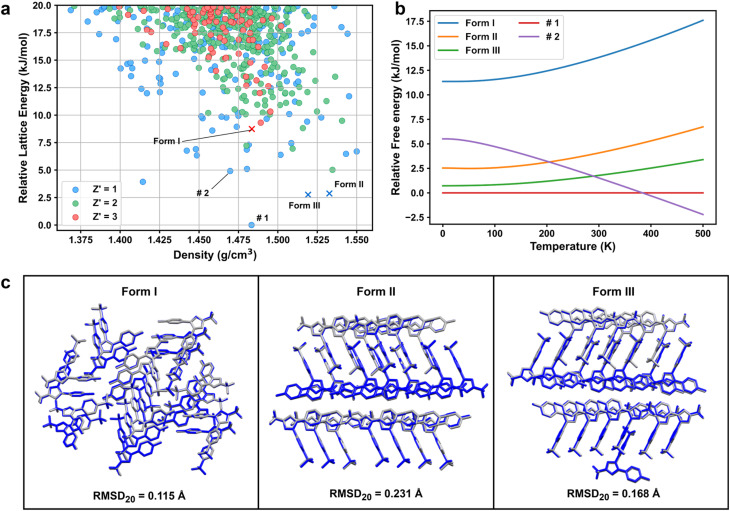
(a) Relative lattice energy landscape at 0 K of celecoxib predicted by AIMNet2. Experimentally known forms are marked with crosses. (b) Temperature-dependent relative free energies of five selected polymorphs, with form #1 taken as the reference. (c) Overlay of experimental crystal structures (gray) and corresponding AIMNet2-optimized structures (blue) for form I (CSD refcode DIBBUL03),^7^ form II (CSD refcode DIBBUL01),^5^ and form III (CSD refcode DIBBUL).^4^ Hydrogen atoms are omitted for clarity.

We assessed the fine-tuned AIMNet2 model on three criteria: identification of known polymorphs in the candidate landscape, accuracy of relative energy ranking, and fidelity of optimized geometries to experiment (Fig. 2). Fig. 2a displays the distribution of candidate crystal structures in the density-relative lattice energy space, revealing that the AIMNet2 model efficiently explores the complex crystal configuration space and identifies thermodynamically stable low-energy structures. In this energy landscape, the structurally known form II and form III are accurately located in regions of low energy, indicating the model's strong polymorph identification capability. In contrast, form I exhibits a significantly higher lattice energy, approximately 8.86 kJ mol^−1^ above form III, consistent with experimental observations that form III is thermodynamically more stable. AIMNet2 predicts form II to lie 0.12 kJ mol^−1^ above form III; this gap is well below the model's energy uncertainty (Table S3), so the predicted ordering should be regarded as consistent with rather than diagnostic of experiment.

To further evaluate the robustness of the AIMNet2 energy ranking, we recalculated the relative lattice energies of the five selected polymorphs with the hybrid functional PBE0 + MBD and B86bPBE50 + XDM (Table S2 and Fig. S4). Although the absolute energy differences vary with the DFT functional and optimized geometry, all approaches consistently predict a shallow energy landscape containing multiple structures within only a few kJ mol^−1^. In particular, polymorphs #1, II, and III remain among the lowest-energy structures across all methods, while polymorph #2 is consistently predicted to lie within approximately 1–5 kJ mol^−1^ of the global minimum.

Fig. 2b presents the temperature-dependent relative free energy profiles of five selected polymorphs, including contributions from zero-point energy (ZPE). Interestingly, after incorporating thermal corrections within the harmonic approximation, two previously unreported low-energy crystal structures candidates (denoted as #1 and #2) are also predicted at 300 K, with free energies 1.78 and 0.27 kJ mol^−1^ lower than form III, respectively, and notably lower densities. These findings suggest the possible existence of undiscovered stable polymorphs. Their structural and conformational characteristics will be further discussed below.

It is important to emphasize that these structures are computational predictions based on thermodynamic considerations and have not been experimentally observed. As widely recognized in crystal structure prediction, low lattice or free energy alone does not guarantee experimental accessibility, as kinetic barriers, nucleation pathways and solvent effects can prevent the formation or isolation of thermodynamically competitive forms.

Nevertheless, the small free-energy differences suggest that these candidates lie within the experimentally relevant energetic window and may become accessible under alternative crystallization conditions, such as melt crystallization or solvent-mediated transformations. As such, they provide physically motivated targets for future experimental screening rather than definitive new polymorph assignments.

Fig. 2b also shows that the free energy difference between form I and form II compared to form III slowly increases with increasing temperature. In contrast, the relative stability of structures #1 and #2 varies more significantly: structure #2, for instance, is less stable than form III at 0 K but becomes more stable above 300 K. This suggests that #2 may possess stability at moderate to high temperatures, warranting further experimental validation.

Fig. 2c presents the 3D overlay of AIMNet2-optimized structures with their corresponding experimental crystal structures. The calculated RMSD_20_ values (root-mean-square displacement of a 20-molecule cluster) are 0.115, 0.231, and 0.168 Å for forms I, II, and III, comfortably below the 0.3 Å threshold typically considered good agreement in CSP benchmarks.

### Elastic properties

Table 1 presents the PBE + MBD and AIMNet2 predictions of the Young's modulus of (001) crystal faces for celecoxib forms I and II at 0 K following full lattice geometry optimization. Similar calculations were also performed for the Young's modulus of (010) face of celecoxib Form III. These crystal faces were predicted to be the dominant morphology facets using the growth morphology approach implemented in BIOVIA Materials Studio.^24^ Both computational methods substantially overestimate the modulus compared to experiment: for form I, the experimental value is 3.18 ± 1.01 GPa, whereas PBE + MBD and AIMNet2 predict 20.7 GPa and 19.1 GPa, respectively; for form II, the experimental value is 16.27 ± 0.43 GPa, compared to 22.7 GPa (PBE + MBD) and 24.6 GPa (AIMNet2).

**Table 1:** Young's modulus of the (001) faces of CEL forms I and II and the (010) face of CEL form III (GPa)

	I	II	III
Exp.	3.18 ± 1.01^7^	16.27 ± 0.43^5^	—
AIMNet2 (0 K)	19.1	24.6	22.4
PBE + MBD (0 K)^a^	20.7	22.7	23.9
AIMNet2 (exp cell)	17.1	16.1	20.7
PBE + MBD (exp cell)^a^	19.8	22.8	20.9
DFTB3 + D4 (exp cell)^b^	13.7	16.7	10.0
PBE + MBD* (exp cell)^c^	—d	18.0	12.4

a Calculated by FHI-aims.

b Calculated by BIOVIA Materials Studio DFTB+.

c Calculated by BIOVIA Materials Studio CASTEP.

d BIOVIA Materials Studio CASTEP PBE + MBD* could not handle this system due to the excessive computational cost.

We do not question the experimental measurements: form II shows good agreement between theory and experiment under the same protocols, supporting their reliability. The discrepancy for form I instead reflects limitations of the underlying physical models. Static-lattice calculations at 0 K neglect thermal expansion and the strong anharmonicity expected in an ultra-soft crystal, both of which lower the effective stiffness measured at 300 K. Quasi-harmonic corrections only partially recover this softening (see below), suggesting that explicit anharmonic treatments are required to describe the macroscopic elastic response of form I.

To validate the effect of thermal lattice expansion, the Young's modulus of three forms were calculated after molecular geometry optimization, with lattice parameters fixed at the SCXRD measurement temperature (130 K, 100 K and 293 K for forms I, II, and III, respectively). From Table 1, it can be seen that AIMNet2 predicts a significant reduction in stiffness for all solid forms with increasing temperature, even though the temperatures considered for forms I and II are still well below ambient. For form II, AIMNet2 yields a Young's modulus of 16.1 GPa, which is in excellent agreement with the experimental value. In contrast, PBE + MBD predicts only a slight decrease in the Young's modulus of form I (20.7 *vs.* 19.8 GPa), while that of form II remains nearly unchanged (22.7 *vs.* 22.8 GPa). The PBE + MBD* calculation performed in BIOVIA Materials Studio^24^ gives a Young's modulus of 18.0 GPa for form II, mildly overestimating the experimental value. Interestingly, DFTB3 + D4 predicts a Young's modulus of 16.7 GPa for form II, which is also in very good agreement with experiment. Experimental values of the Young's modulus for CEL form III are not available to validate the predictions.

However, although reasonable agreement with experiment is obtained for form II at certain levels of theory, calculations at all levels fail to reproduce the much lower stiffness observed for form I. As such, celecoxib form I therefore constitutes a stringent benchmark for finite-temperature anharmonic treatments of elastic properties in molecular crystals. In particular, to enable more accurate comparisons between experimental and theoretical results, future work should incorporate temperature effects, for example by employing the Quasi-Harmonic Approximation (QHA) or MD simulations, to capture the temperature-dependent behavior of Young's modulus.

### Effect of thermal expansion on polymorph stability

**Fig. 3:**
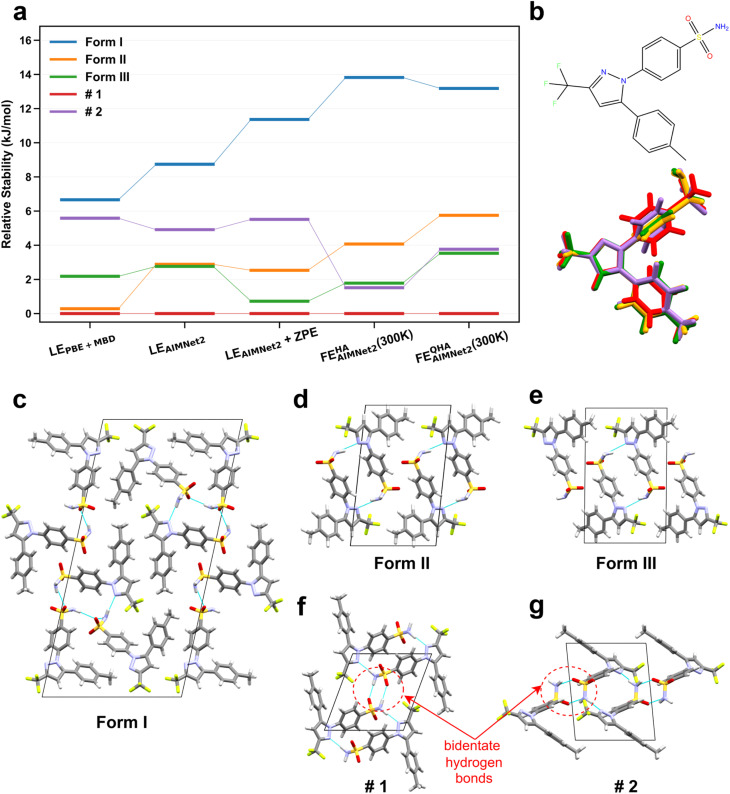
(a) Relative stability ranking of five selected polymorphs of celecoxib. Lattice energies (LE) calculated with PBE + MBD and AIMNet2, AIMNet2 with zero-point energy (ZPE) corrections, and AIMNet2 free energies (FE) calculated using the HA and QHA at 300 K. (b) Overlay of molecular conformations in four *Z*′ = 1 celecoxib polymorphs, with colors corresponding to those in panel (a). (c)–(g) Crystal packing diagrams of the five selected polymorphs.

To account for thermal expansion effects, we performed QHA calculations at 300 K using AIMNet2 for five selected polymorphs. The resulting relative stabilities are shown in Fig. 3a. Without thermal corrections, AIMNet2 predicts that form II and form III are very close in energy, whereas PBE + MBD suggests that form II is more stable and has a similar free energy to polymorph #1. PBE0 + MBD calculations (Table S2 and Fig. S4) yield the same qualitative conclusion that forms II, III, and polymorph #1 belong to a narrow low-energy manifold, supporting the existence of several thermodynamically competitive polymorphs. When thermal corrections within the harmonic approximation are included, the free energy difference (FE_AIMNet2_^HA^(300 K)) between form II and form III increases, with form III becoming comparable in stability to polymorph #2. Further incorporation of thermal expansion *via* QHA (FE_AIMNet2_^QHA^(300 K)) has little impact on the overall energy ranking predicted by AIMNet2, with only a slight reordering between form III and polymorph #2, whose free energy difference is just 0.23 kJ mol^−1^.

Fig. 3c–g illustrate the crystal packing patterns of the five polymorphs. Notably, polymorphs #1 and #2 exhibit intermolecular bidentate hydrogen bonding, while the hydrogen bonding motifs in form II and form III are very similar. This similarity may explain the frequent presence of form II impurities in experimentally obtained form III crystal samples.^3^Fig. 3b further compares the molecular conformations of the four *Z*′ = 1 polymorphs, revealing that the primary differences arise from the torsional angles of the pyrazole ring relative to the sulfonylphenyl and *p*-tolyl groups, as well as the orientation of the sulfonamide –NH_2_ group about the S–N bond.

## Conclusions and outlook

In this study, we developed a comprehensive CSP workflow powered by the AIMNet2 MLIP to investigate the polymorphism of celecoxib. By integrating GPU-accelerated crystal structure generation, energy ranking, and property prediction, our workflow not only reproduces the experimental stability hierarchy of known polymorphs but also predicts two energetically competitive crystal structure candidates that have not yet been experimentally reported. The predicted free energy differences between these candidates and the established form III are within 4 kJ mol^−1^, placing them within the typical uncertainty range relevant for pharmaceutical polymorphism. While their experimental existence cannot be claimed on the basis of computation alone, the distinct packing motifs and hydrogen-bonding arrangements identified here provide concrete structural hypotheses that may guide targeted experimental screening and potentially impart favorable physicochemical properties, such as enhanced solubility or mechanical flexibility.

We have shown that an AIMNet2 model fine-tuned *via* cluster-based active learning recovers the experimental energy hierarchy of celecoxib polymorphs, reproduces unit cell parameters within ∼1.5% and RMSD_20_ below 0.25 Å, and predicts two previously unreported low-energy structures (#1 and #2) within 4 kJ mol^−1^ of the most stable known form III at 300 K. Hybrid-DFT calculations place forms II, III, #1, and #2 within a narrow low-energy window, indicating the presence of several thermodynamically competitive polymorphs. Low predicted free energy does not by itself guarantee experimental accessibility: kinetic barriers, nucleation pathways, solvent effects, and crystallizability can prevent the formation of thermodynamically competitive forms. The candidates should therefore be read as physically motivated targets for experimental polymorph screening, not as discovered new forms.

The form I Young's modulus discrepancy delineates a clear methodological limitation: static-lattice MLIP + QHA cannot capture the strong anharmonicity that governs ultra-soft elastic response. Future work should incorporate explicit anharmonic methods such as self-consistent phonons or MD-derived elastic constants. Looking forward, experimental validation of the predicted celecoxib candidates through solvent-mediated transformations or melt crystallization would directly test the framework. The cluster-active-learning protocol developed here is broadly transferable to other flexible APIs and could shorten the timescale on which polymorphism is mapped during early-stage drug development.

## Author contributions

P. Z.: methodology, investigation, visualization, writing – original draft. Y. A.: investigation, writing – original draft. C. S.: writing – review & editing. O. I.: conceptualization, supervision, writing – review & editing.

## Conflicts of interest

The authors declare no competing interests.

## Supplementary Material

SC-017-D5SC09784C-s001

## Data Availability

The AIMNet2 model and the optimized crystal structures are available at https://github.com/isayevlab/csp-celebrex. Supplementary information (SI): crystal structure generation, training data preparation, AIMNet2 model training, QM calculations, and the computation of free energies and elastic constants. See DOI: https://doi.org/10.1039/d5sc09784c.
