# Supplementary material for: Exploring celecoxib polymorph landscape using AIMNet2 machine learning interatomic potential
Source: Chem Sci. 2026 Jun 10. Online ahead of print. doi: 10.1039/d5sc09784c (PMC13284824; doi:10.1039/d5sc09784c)
Supplement: SC-OLF-D5SC09784C-s001 [file SC-OLF-D5SC09784C-s001.pdf]

Supplementary Information (SI) for Chemical Science.
This journal is © The Royal Society of Chemistry 2026

Supporting Information:

Exploring Celecoxib Polymorph Landscape Using AIMNet2 Machine Learning Interatomic Potential

Peikun Zheng[1], Yuriy A. Abramov[2,3], Changquan Calvin Sun[4], Olexandr Isayev[1]*

[1]*Department of Chemistry, Carnegie Mellon University, Pittsburgh, Pennsylvania 15213, United States*

[2]Porton USA | J-Star Research Inc, Cranbury, NJ 08512, United States

[3]Eshelman School of Pharmacy, University of North Carolina, Chapel Hill, NC 27599, *United States*

[4] *Department of Industrial and Molecular Pharmaceutics, Purdue University, 124C RHPH, 575 Stadium Mall Drive, West Lafayette, IN 47907*

E-mail: olexandr@olexandrisayev.com

Crystal structure generation

The generation of crystal structures is a crucial step in crystal structure prediction. However, most crystal structure generation programs still suffer from some limitations. These programs typically rely on a trial-and-error approach to generate potential structures, which is inherently inefficient and often necessitates numerous iterations to achieve success, potentially failing even after reaching the maximum number of attempts. Furthermore, many programs require larger volumes to reduce the likelihood of unphysically short atomic contacts in the generated structures. The majority of these programs are constrained to scenarios where Z' equals 1 (i.e., the unit cell contains only one symmetrically independent molecule), thereby limiting their effectiveness in generating more complex structures with multiple independent molecules. This restriction significantly hinders their application in the generation of high Z' and cocrystal crystal structures.

To address these challenges and expedite the generation of a large number of crystal structures, we have implemented a GPU-accelerated crystal structure generation code based on PyTorch. This implementation significantly improves both the efficiency and diversity of structure generation, facilitating a comprehensive exploration of potential candidate structures and substantially accelerating the investigation of various crystal packing patterns. The code currently supports all 230 space groups, but only general Wyckoff positions are implemented at this stage.

Figure S1 presents the flowchart of the crystal structure generation process. In the first step, we need to provide the molecular coordinates, the number of molecules in the asymmetric unit cell (Z'), the number of symmetry operations (Z), and the initial volume of the crystal. Based on this information, the program generates a random unit cell that meets these criteria. Then it places the input molecule at random positions within the unit cell, generating all symmetry-equivalent molecules according to the corresponding space group's symmetry operations. If any unreasonable short atomic contacts are detected in the generated structure, the program performs rigid-body relaxation to obtain a physically reasonable structure, using a criterion greater than the sum of the atomic van der Waals radii.

Throughout the generation process, we leverage PyTorch's tensor computation capabilities to implement batch operations, fully exploiting the parallel computing power of GPUs, enabling the program to generate a large number of candidate crystal structures simultaneously in a single run, rather than sequentially.

To systematically investigate the polymorphism of Celecoxib, we generated 50k, 50k, and 200k initial crystal structures for $Z' = 1$, 2, and 3, respectively.

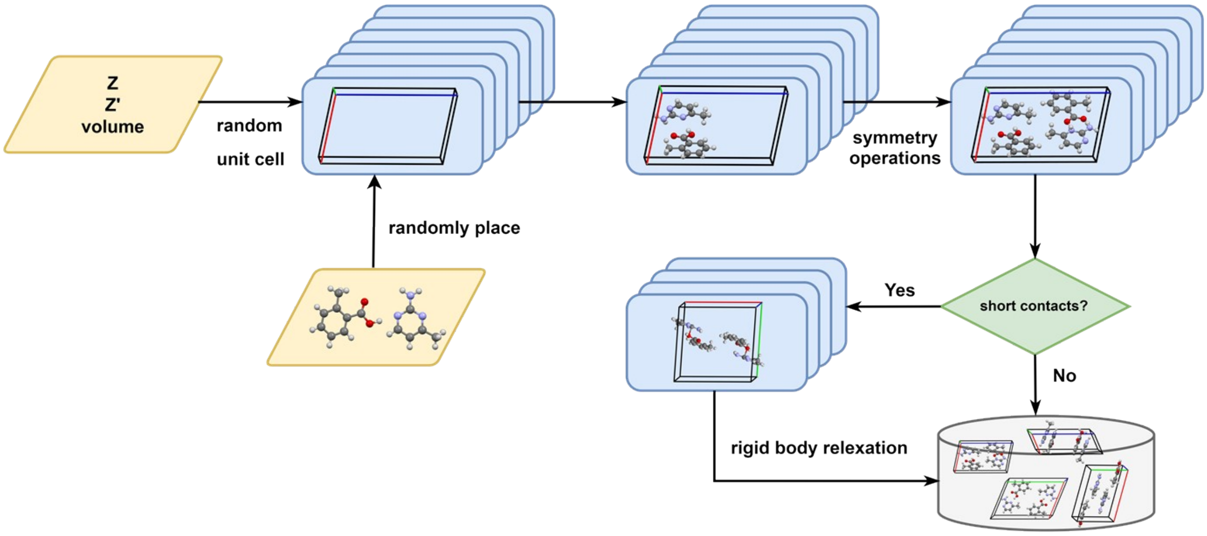

Figure S1. The workflow of crystal structure generation.

Training data generation

We first generated densely sampled conformations based on the SMILES representation of Celecoxib using OpenEye Omega[1]. The sampled conformations were subsequently subjected to 25 ps of restrained molecular dynamics (MD) simulations at 300 K employing the pre-trained AIMNet2 model[2] to thoroughly explore the conformational space. All conformers were then optimized with a force convergence criterion of 0.01 eV/Å, followed by conformational deduplication using RMSD calculations based on the Kabsch algorithm[3,4], using a threshold of 0.5 Å. Ultimately, monomer structures within 10 kcal/mol of the lowest energy conformer were retained.

By randomly combining these optimized monomers, we progressively constructed dimers, trimers, and tetramers, applying the same optimization and screening procedures. For each molecular cluster, including the monomers, a short MD simulation of 0.5 ps was performed at 500 K, with configurations saved every 0.1 ps, yielding five configurations per trajectory. QM calculations were then performed on all generated molecular clusters (hereafter referred to as N-mers), resulting in an initial N-mers dataset (N = 1-4) comprising 18,816 structures.

Model training

We employed a transfer learning strategy by initializing from four pre-trained AIMNet2 models and further trained them on the N-mers dataset to obtain four Celecoxib-specific models. Our tests show that transfer learning yields lower errors for both energies and forces compared to training from scratch (Figure S2). Model training was driven by a multi-task loss function that jointly optimizes total energy, atomic forces, Hirshfeld atomic charges, and the atomic C_6 dispersion coefficients and polarizabilities from the D4 dispersion scheme[5,6], with respective loss weights of 1.0, 0.2, 0.5, 0.5, and 0.5.

To accurately describe dispersion interactions, we introduced a hybrid explicit-implicit dispersion correction scheme. During inference, the model-predicted atomic C_6 coefficients and polarizabilities were used to explicitly compute pairwise D4 dispersion energies via the Tkatchenko-Scheffler method[7]. Validation shows that these two-body energies agree closely with standard D4 results. During training, the three-body D4 correction term was implicitly learned by incorporating it into the energies and forces of the training data. This hybrid correction strategy enables a comprehensive description of both two-body and three-body dispersion interactions in molecular crystals while maintaining the computational efficiency of the AIMNet2, thus providing a more accurate and reliable potential energy surface foundation for subsequent crystal structure prediction.

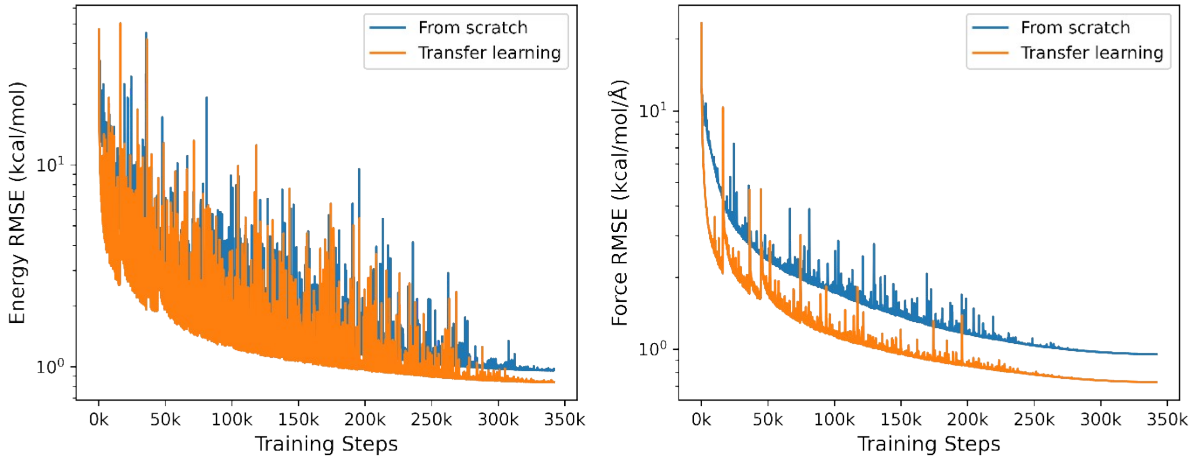

Figure S2. Comparison of energy and force learning curves between models trained from scratch and via transfer learning.

Active learning

The overall workflow of the active learning procedure is illustrated in Figure S2. First, we optimized the candidate crystal structures using one of the four fine-tuned AIMNet2 models described above to construct a comprehensive energy landscape. From this landscape, structures with relative lattice energies lower than 15 kJ/mol were selected, and molecular clusters (*N*-mers) were extracted from these low-energy structures. To capture potential non-equilibrium conformations, we performed short MD simulations at 500 K for 1 ps on the extracted N-mers.

Subsequently, we evaluated both the energy uncertainty and force uncertainties of each structure using an ensemble of four AIMNet2 models:

$$\sigma_E = \frac{1}{N_m}\sqrt{\frac{1}{M}\sum_{m=1}^{M}\left(E^{(m)} - \bar{E}\right)^2} \tag{1}$$

$$\sigma_F = \sqrt{\frac{1}{N_a}\sum_{i=1}^{N}\frac{1}{M}\sum_{m=1}^{M}\left\|\mathbf{F}_i^{(m)} - \bar{\mathbf{F}}_i\right\|^2} \tag{2}$$

where M is the number of models, and N_m and N_a are the numbers of molecules and atoms in a given structure, respectively. If a structure exhibited an energy uncertainty σ_E exceeding 1 kJ/mol or a force uncertainty σ_F exceeding 1 kcal/mol/Å, it was considered to have high prediction uncertainty. These high-uncertainty structures were then forwarded for QM calculations and incorporated into the training dataset to further refine the AIMNet2 models, thereby improving their accuracy and robustness. The active learning procedure was deemed converged when the fraction of uncertain candidates fell below a predefined threshold (1% in this work).

We performed two rounds of active learning. In the first round, we extracted 1- to 4-mers and added 3,354 new structures; in the second round, we expanded the pool to 1- to 6-mers, adding 222 structures. The detailed size distribution of training N-mers for each active learning cycle is summarized in Table S1. By selectively augmenting the training set with these representative, high-uncertainty structures, we significantly improved the model's generalization and predictive performance. This active learning strategy not only reduced computational cost but also accelerated model convergence, thereby substantially enhancing the overall efficiency and reliability of molecular crystal structure prediction.

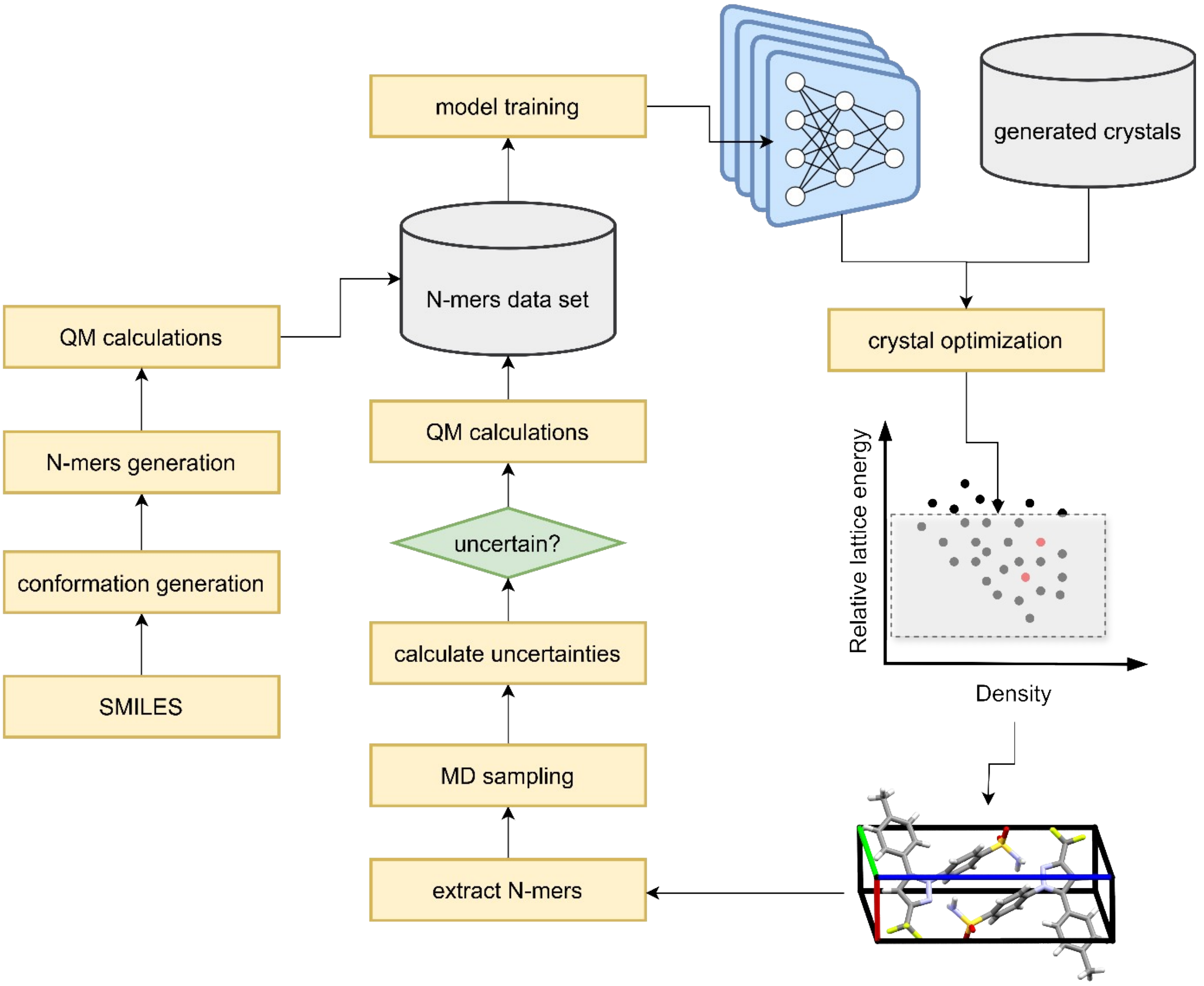

Figure S3. The workflow of training AIMNet2.

Table S1. Size distribution of the sampled and high-uncertainty N-mers in each active learning cycle.

N-mers	AL = 0	AL = 1			AL = 2		
	N_{tot}	N_{tot}	N_{unc}	N_{unc} (%)	N_{tot}	N_{unc}	N_{unc} (%)
1	270	168	0	0.00	219	0	0.00
2	2694	1393	54	3.88	1720	0	0.00
3	12798	10476	390	3.72	12655	6	0.05
4	3054	48851	2910	5.96	59138	18	0.03
5	—	—	—	—	184632	138	0.07
6	—	—	—	—	427427	60	0.01
Total	18816	60888	3354	5.51	685791	222	0.03

QM calculation

We performed gas-phase QM calculations for the molecules in the *N*-mers dataset using the ORCA 6.0 software[8,9] at the PBE/def2-TZVP[10,11] level of theory. D4 dispersion corrections were carried out using the DFT-D4 program, which also provided the atomic C_6 dispersion coefficients and polarizabilities. Periodic DFT calculations were conducted with the FHI-aims program[12]. We employed the PBE functional with the many-body dispersion interactions (MBD) at the non-local level[13] and used the light species basis settings for geometry optimization. After geometry optimization, single-point energy calculations were performed using the tight species basis settings with PBE+MBD and B86bPBE+XDM to ensure reliable energy evaluation accuracy. For the hybrid functional calculations (PBE0+MBD and B86bPBE50+XDM), the mixed-basis approach[14,15] was employed to maintain computational tractability. The resulting relative lattice energies are summarized in Figure S4 and Table S2.

For the selection of the *k*-point grid, we ensured that the product of the number of *k*-points (n) in each direction and the unit cell length (a) satisfied $n \times a \geqslant 25$ Å to achieve sufficient *k*-space sampling density. The convergence criterion for atomic forces in periodic DFT geometry optimizations was set to a root-mean-square (RMS) force threshold of 0.005 eV/Å. Given the high computational efficiency of AIMNet2 in predicting energies and forces, a more stringent convergence criterion of 0.002 eV/Å was adopted for structure optimizations using AIMNet2.

Table S2. Comparison of relative lattice energies (kJ/mol) calculated using different methods, basis sets, and optimized geometries.

Method	I	II	III	# 1	# 2
AIMNet2 [a]	8.74	2.87	2.76	0.0	4.91
PBE + MBD/tight [b]	6.66	0.28	2.18	0.0	5.58
PBE0 + MBD/tight [b]	5.08	-2.01	-0.01	0.0	2.67
PBE + MBD/tight [a]	5.49	1.73	1.90	0.0	5.26
PBE0 + MBD/tight [a]	3.13	-0.65	-0.01	0.0	3.06
PBE0 + MBD/mixed [a]	3.19	-1.82	-0.26	0.0	2.95
B86bPBE + XDM/tight [a]	2.75	-1.59	-1.46	0.0	4.85
B86bPBE50 + XDM/mixed [a]	-0.63	-6.38	-3.92	0.0	0.98

[a] Calculations based on AIMNet2-optimized geometries.

[b] Calculations based on PBE+MBD/light-optimized geometries.

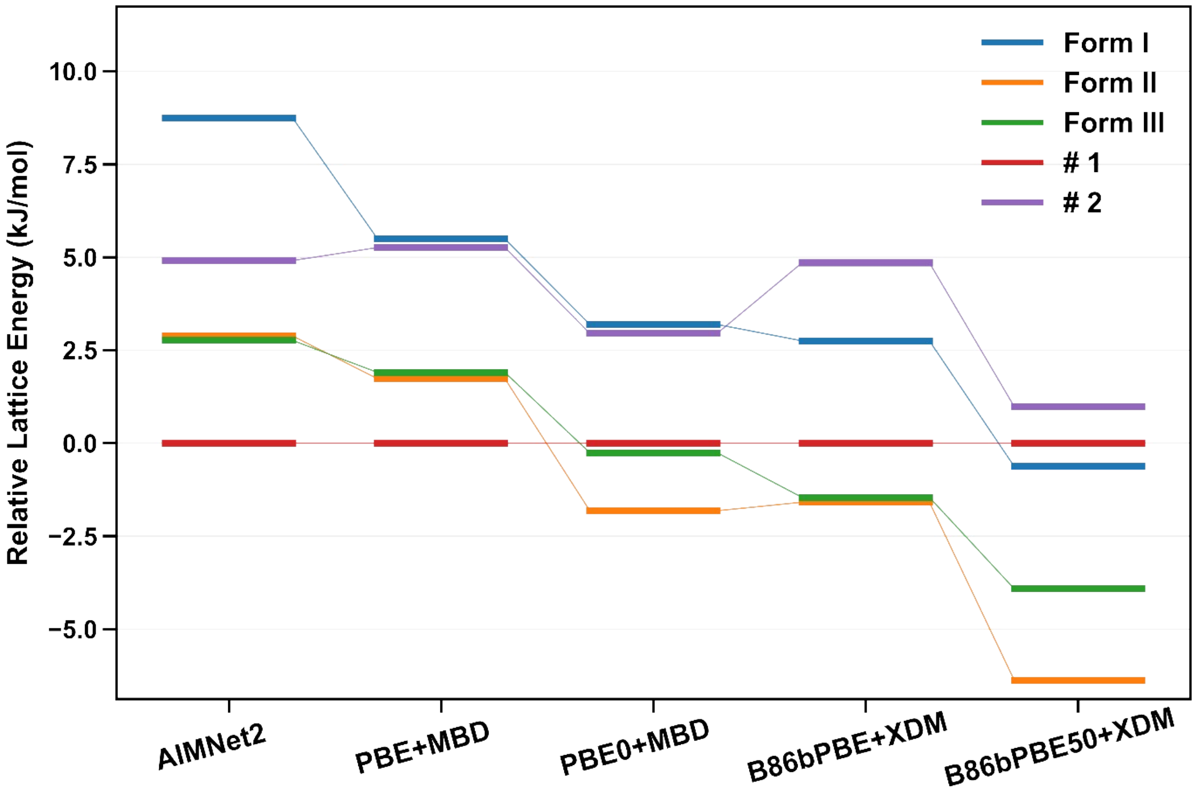

Figure S4. Relative lattice energy rankings of five selected polymorphs of Celecoxib based on AIMNet2-optimized geometries. Results obtained with the PBE0 and B86bPBE50 functionals using the mixed basis set approach are shown.

Free energy and elastic constant calculations

The Helmholtz free energy calculations were computed by interfacing AIMNet2 with the Phonopy package[16] to obtain temperature-dependent thermodynamic properties of crystals. In the QHA calculations, 11 structures were generated by scaling the optimized geometry with scale factors ranging from 0.95 to 1.05 in increments of 0.05. Each structure was then re-optimized with fixed lattice parameters, and the resulting energy-volume data were fitted using the Birch-Murnaghan equation of state[17].

Elastic constants were calculated using the stress-strain method implemented via an interface with pymatgen[18], applying strain values of -0.003, -0.001, 0.001, and 0.003. The DFT calculations in FHI-aims for elastic constant evaluations were performed using the light settings due to the high computational cost associated with elastic tensor calculations.

Elastic constant calculations with BIOVIA DFTB+ and CASTEP software

BIOVIA Materials Studio[19] DFTB+[20] and CASTEP[21] modules were used for mechanical property predictions of forms I, form II and III, with unit cell parameters fixed at their crystallographic values.

Geometry optimization for CEL forms II and III were performed at the DFTB+D4 level of theory using the 3ob parameter set and Grimme D4 dispersion correction[5,6], adopting "Fine" quality settings. This was followed by mechanical property predictions at the same level of theory using the stress-strain method (referred to as the *constant strain* method in Materials Studio). Similar calculations were carried out for CEL form I at the DFTB+D42B level, employing the Grimme D4 dispersion correction with two-body terms only. Similar calculations were carried out for CEL form I at the DFTB+D42B level, employing the Grimme D4 dispersion correction with two-body terms only.

BIOVIA CASTEP calculations for CEL form II were performed at the PBE+MBD*[10,13,22] level of theory with "Fine" accuracy settings, except that the plane-wave energy cutoff was slightly reduced to 550 eV. After molecular geometry optimization, mechanical properties were evaluated at the same level using the stress-strain method. Due to very poor convergence, the "Medium" accuracy level was adopted for CASTEP calculations of the elastic constants of form III, while maintaining the same 550 eV plane-wave cutoff. Because of the high computational cost, CASTEP calculations were not feasible for CEL form I.

Model evaluation

To evaluate the generalization performance of the model on unseen data, we constructed a fully independent held-out test set that did not participate in either training or the active learning procedure. Specifically, 20 systems were randomly selected from the N-mer (N = 2-6) configurations included in the training set. For each system, a short MD simulation of 1 ps was performed at 500 K, and configurations were saved every 0.1 ps, yielding 10 configurations per trajectory. All configurations generated in this manner were excluded from model training and active learning. In total, this procedure resulted in a held-out test set comprising 1000 systems, which was used to quantitatively evaluate the accuracy of energy and force predictions.

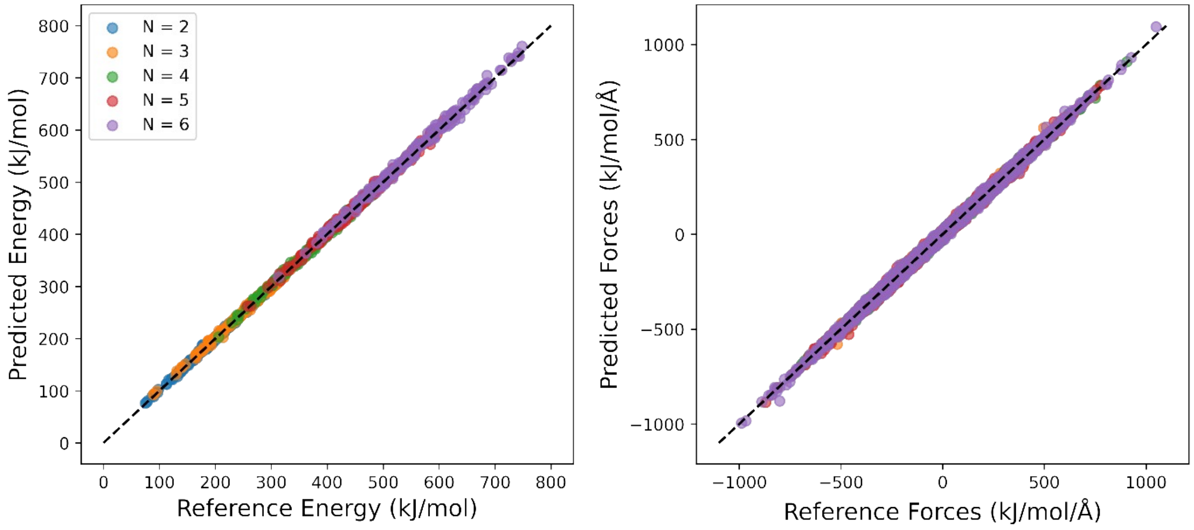

Figure S5. Correlation between AIMNet2-predicted energies and forces and the corresponding reference DFT values for the held-out test set.

Table S3. Performance metrics of the AIMNet2 model for each N-mer (N = 2–6), evaluated on a held-out molecular cluster test set of celecoxib. Energies are reported in kJ/mol and forces in kJ/mol/Å.

N-mers	Energy				Force	
	MAE	MAE / N	RMSE	RMSE / N	MAE	RMSE
2	2.10	1.05	2.76	1.38	2.74	3.89
3	2.89	0.96	3.66	1.22	2.88	4.10
4	3.62	0.91	4.47	1.12	3.01	4.26
5	4.58	0.92	5.73	1.15	3.29	4.69
6	5.56	0.93	6.77	1.13	3.39	4.82

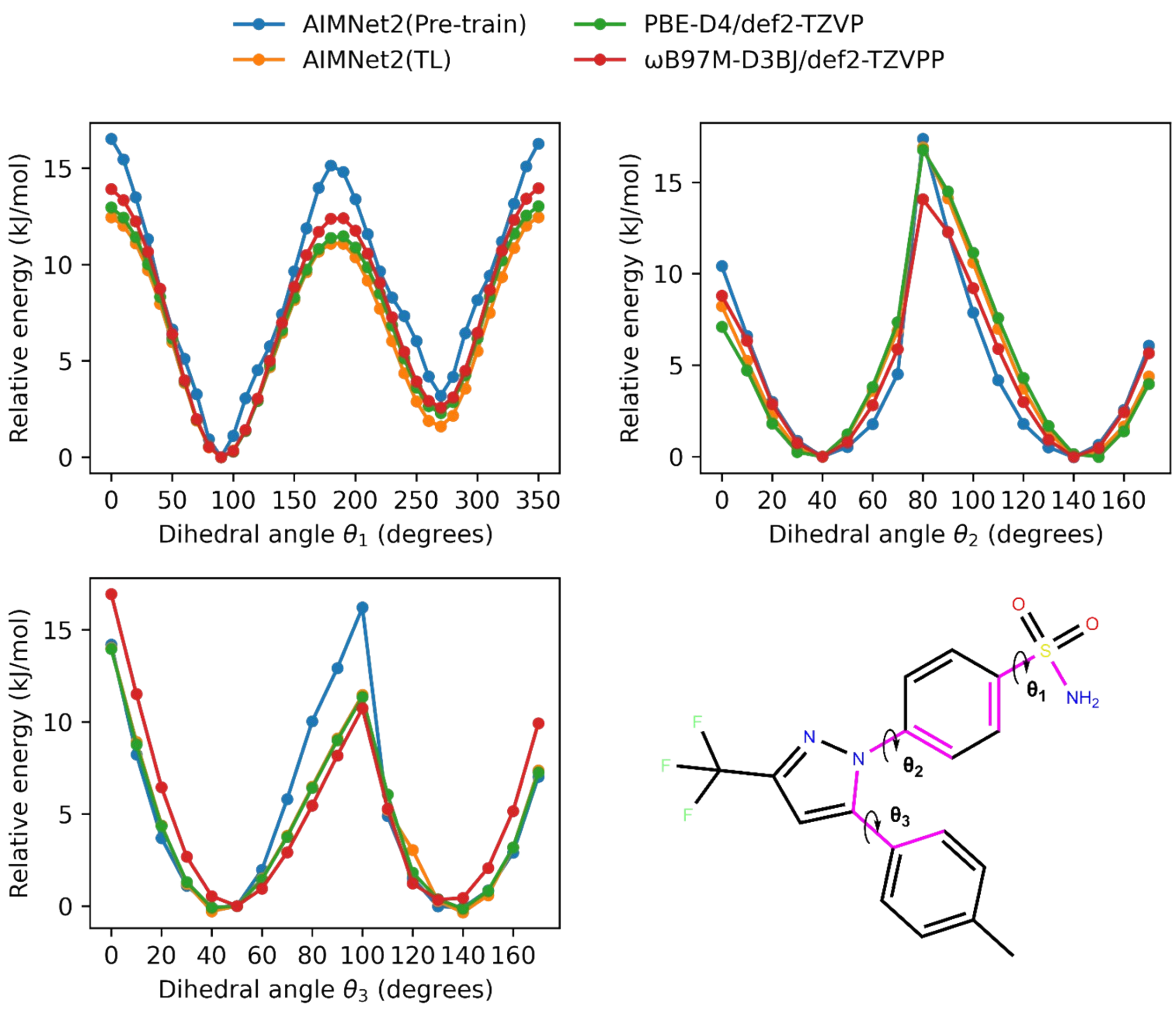

Figure S6. Conformational energy profiles for the rotation of three key dihedral angles in the celecoxib molecule.

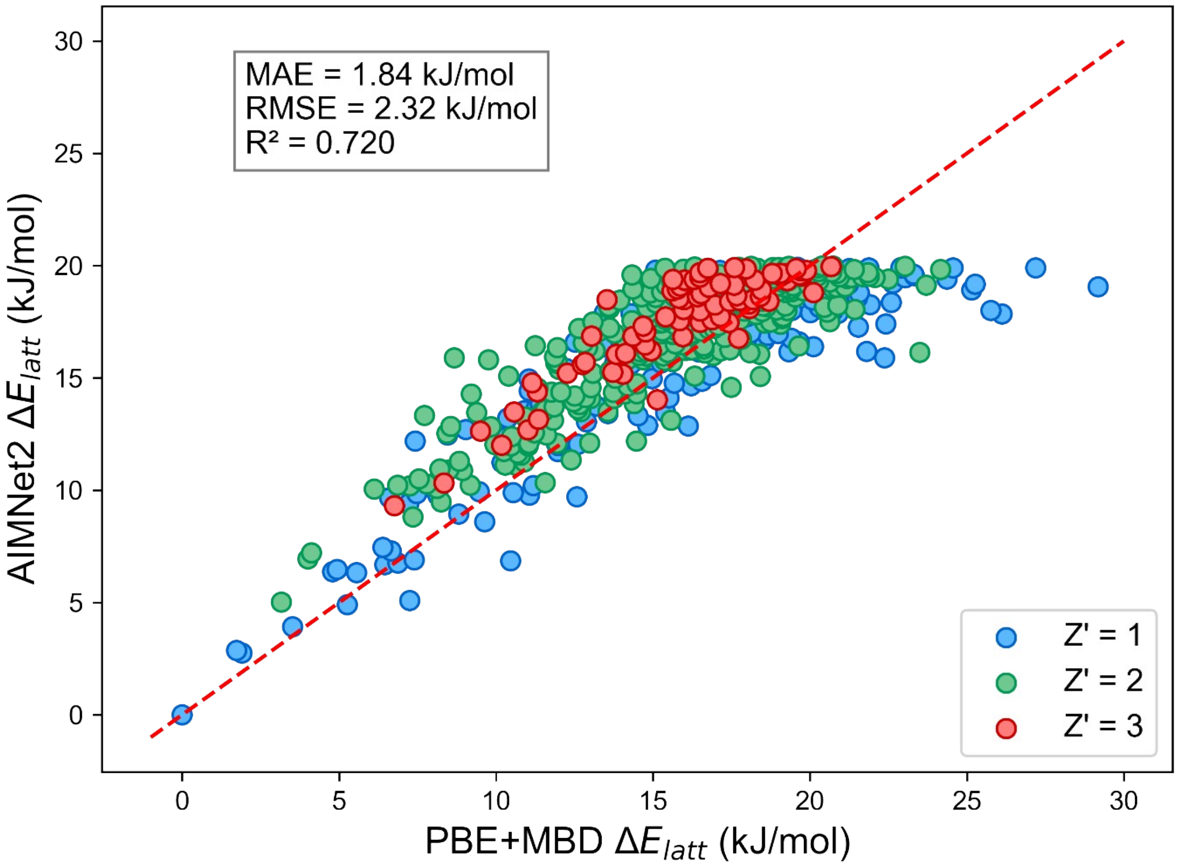

Figure S7. Correlation between relative lattice energies predicted by AIMNet2 and PBE + MBD, evaluated on AIMNet2-optimized geometries.

Table S4. Crystal structures optimized with AIMNet2 and PBE + MBD relative to experimental structures. Relative errors (%) for cell lengths (*a*, *b*, *c*), angles (*α*, *β*, *γ*), cell volume, and density are reported, along with $RMSD_{20}$ values (Å).

Form	Method	*a*	*b*	*c*	*α*	*β*	*γ*	Volume	Density	$RMSD_{20}$
I	AIMNet2	1.39	-0.11	-0.50	0.03	-0.32	-0.03	0.82	-0.80	0.115
	PBE+MBD	-0.44	-1.05	-0.72	-0.78	0.78	0.70	-2.20	2.21	0.160
II	AIMNet2	-0.18	2.06	-0.21	-1.21	-2.23	-1.27	1.04	-1.03	0.231
	PBE+MBD	0.14	-0.93	-0.71	-0.51	0.84	1.37	-1.11	1.10	0.144
III	AIMNet2	0.46	0.36	-0.81	-1.49	-0.04	0.27	0.22	-0.20	0.168
	PBE+MBD	0.04	-2.33	-1.29	-1.18	0.00	-1.26	-2.90	3.02	0.196

Table S5. Crystal structures optimized using AIMNet2 and PBE+MBD for two crystals forms (#1 and #2). Cell lengths (*a*, *b*, *c*) are reported in Å, angles (*α*, *β*, *γ*) in degrees, cell volumes in Å^3, densities in g/cm^3.

Form	Method	*a*	*b*	*c*	*α*	*β*	*γ*	Volume	Density
#1	AIMNet2	9.377	10.081	10.440	103.95	106.69	105.70	853.88	1.483
	PBE+MBD	9.169	10.256	10.540	100.94	112.27	105.52	835.39	1.516
#2	AIMNet2	8.388	9.307	11.711	82.31	73.45	81.03	861.77	1.470
	PBE+MBD	8.189	9.271	11.628	82.10	74.02	81.76	835.49	1.516

Table S6. Computational time for each task in the crystal structure prediction workflow. The DFT calculation time is reported in CPU hours, while all other tasks are reported in GPU hours.

Task	Compute time (hours)
Crystal generation	75
DFT calculation	30086
AIMNet2 training	182
Crystal optimization	1028

References

(1) Hawkins, P. C. D.; Skillman, A. G.; Warren, G. L.; Ellingson, B. A.; Stahl, M. T. Conformer Generation with OMEGA: Algorithm and Validation Using High Quality Structures from the Protein Databank and Cambridge Structural Database. *J. Chem. Inf. Model.* **2010**, *50* (4), 572–584.

(2) Anstine, D. M.; Zubatyuk, R.; Isayev, O. AIMNet2: A Neural Network Potential to Meet Your Neutral, Charged, Organic, and Elemental-Organic Needs. *Chem. Sci.* **2025**, *16* (23), 10228–10244.

(3) Kabsch, W. A Solution for the Best Rotation to Relate Two Sets of Vectors. *Acta Crystallogr. A: Cryst. Phys. Diffr. Theor. Gen. Crystallogr.* **1976**, *32* (5), 922–923.

(4) Kabsch, W. A Discussion of the Solution for the Best Rotation to Relate Two Sets of Vectors. *Acta Crystallogr. A: Cryst. Phys. Diffr. Theor. Gen. Crystallogr.* **1978**, *34* (5), 827–828.

(5) Caldeweyher, E.; Bannwarth, C.; Grimme, S. Extension of the D3 Dispersion Coefficient Model. *J. Chem. Phys.* **2017**, *147* (3), 034112.

(6) Caldeweyher, E.; Ehlert, S.; Hansen, A.; Neugebauer, H.; Spicher, S.; Bannwarth, C.; Grimme, S. A Generally Applicable Atomic-Charge Dependent London Dispersion Correction. *J. Chem. Phys.* **2019**, *150* (15), 154122.

(7) Tkatchenko, A.; Scheffler, M. Accurate Molecular Van Der Waals Interactions from Ground-State Electron Density and Free-Atom Reference Data. *Phys. Rev. Lett.* **2009**, *102* (7), 073005.

(8) Neese, F. The ORCA Program System. *WIREs Comput. Mol. Sci.* **2012**, *2* (1), 73–78.

(9) Neese, F. Software Update: The ORCA Program System—Version 6.0. *WIREs Comput. Mol. Sci.* **2025**, *15* (2), e70019.

(10) Perdew, J. P. Generalized Gradient Approximation Made Simple. *Phys. Rev. Lett.* **1996**, *77* (18), 3865.

(11) Weigend, F.; Ahlrichs, R. Balanced Basis Sets of Split Valence, Triple Zeta Valence and Quadruple Zeta Valence Quality for H to Rn: Design and Assessment of Accuracy. *Phys. Chem. Chem. Phys.* **2005**, *7* (18), 3297–3305.

(12) Blum, V.; Gehrke, R.; Hanke, F.; Havu, P.; Havu, V.; Ren, X.; Reuter, K.; Scheffler, M. *Ab Initio* Molecular Simulations with Numeric Atom-Centered Orbitals. *Comput. Phys. Commun.* **2009**, *180* (11), 2175–2196.

(13) Tkatchenko, A. Accurate and Efficient Method for Many-Body van Der Waals Interactions. *Phys. Rev. Lett.* **2012**, *108* (23).

(14) Price, A. J. A.; Mayo, R. A.; Otero-de-la-Roza, A.; Johnson, E. R. Accurate and Efficient Polymorph Energy Ranking with XDM-Corrected Hybrid DFT. *CrystEngComm* **2023**, *25* (6), 953–960.

(15) A. Price, A. J.; Otero-de-la-Roza, A.; R. Johnson, E. XDM-Corrected Hybrid DFT with Numerical Atomic Orbitals Predicts Molecular Crystal Lattice Energies with Unprecedented Accuracy. *Chem. Sci.* **2023**, *14* (5), 1252–1262.

(16) Togo, A.; Tanaka, I. First Principles Phonon Calculations in Materials Science. *Scr. Mater.* **2015**, *108*, 1–5.

(17) Birch, F. Finite Elastic Strain of Cubic Crystals. *Phys. Rev.* **1947**, *71* (11), 809–824.

(18) Ong, S. P.; Richards, W. D.; Jain, A.; Hautier, G.; Kocher, M.; Cholia, S.; Gunter, D.; Chevrier, V. L.; Persson, K. A.; Ceder, G. Python Materials Genomics (Pymatgen): A Robust, Open-Source Python Library for Materials Analysis. *Comput. Mater. Sci.* **2013**, *68*, 314–319.

(19) BIOVIA Materials Studio, Release 2025. *San Diego: Dassault Systemes, 2025.*

(20) Hourahine, B.; Aradi, B.; Blum, V.; Bonafé, F.; Buccheri, A.; Camacho, C.; Cevallos, C.; Deshaye, M. Y.; Dumitrică, T.; Dominguez, A.; Ehlert, S.; Elstner, M.; van der Heide, T.; Hermann, J.; Irle, S.; Kranz, J. J.; Köhler, C.; Kowalczyk, T.; Kubař, T.; Lee, I. S.; Lutsker, V.; Maurer, R. J.; Min, S. K.; Mitchell, I.; Negre, C.; Niehaus, T. A.; Niklasson, A. M. N.; Page, A. J.; Pecchia, A.; Penazzi, G.; Persson, M. P.; Řezáč, J.; Sánchez, C. G.; Sternberg, M.; Stöhr, M.; Stuckenberg, F.; Tkatchenko, A.; Yu, V. W. -z.; Frauenheim, T. DFTB+, a Software Package for Efficient Approximate Density Functional Theory Based Atomistic Simulations. *J. Chem. Phys.* **2020**, *152* (12), 124101.

(21) Clark, S. J.; Segall, M. D.; Pickard, C. J.; Hasnip, P. J.; Probert, M. I. J.; Refson, K.; Payne, M. C. First Principles Methods Using CASTEP. *Z. Krist. - Cryst. Mater.* **2005**, *220* (5–6), 567–570.

(22) Ambrosetti, A.; Reilly, A. M.; DiStasio, R. A., Jr.; Tkatchenko, A. Long-Range Correlation Energy Calculated from Coupled Atomic Response Functions. *J. Chem. Phys.* **2014**, *140* (18), 18A508.
